# The causal association between asthma and the risk of frailty: A two-sample Mendelian randomization study

**DOI:** 10.1007/s40520-024-02906-4

**Published:** 2024-12-27

**Authors:** Pingping Ning, Xin Mu, Xingzhi Guo, Rong Zhou, Ge Tian, Rui Li

**Affiliations:** 1 Department of Geriatric Neurology, Shaanxi Provincial People’s Hospital, No.256, Youyi West Road, Xi’an 710068, China; 2 Shaanxi Provincial Clinical Research Center for Geriatric Medicine, Xi’an 710068, People’s Republic of China; 3 Institute of Medical Research, Northwestern Polytechnical University, Xi’an 710068, People’s Republic of China; 4 Department of Neurology, Chengdu First People’s Hospital, No. 18 Wanxiang North Road, Chengdu, 610041 China

**Keywords:** Asthma, Frailty, Mendelian randomization, Causal effect

## Abstract

**Background:**

The correlation between asthma and frailty is increasingly garnering attention. The association between asthma and frailty remains inconclusive in observational studies, and the causality of this relationship still needs to be established.

**Aims:**

Therefore, we employed two-sample Mendelian randomization analyses using genetic instruments to determine the causal association of asthma on frailty.

**Methods:**

Two-sample Mendelian randomization (MR) analyses were performed to assess the causal effect of asthma on frailty. The genetic variants strongly associated with asthma (*P* < 5E-08) during the discovery and replication stages were derived from a recent meta-analysis of genome-wide association studies (GWAS) (N = 408,442) in the UK Biobank and a GWAS in the FinnGen Consortium (N = 217,421), respectively. Summary statistics of the frailty index (N = 175,226) are derived from the latest released GWAS dataset on frailty index. The inverse variance weighted (IVW) method was employed as the primary approach for calculating estimated values, with additional sensitivity analyses and heterogeneity analyses utilized to further validate the results.

**Results:**

Using the IVW method, genetic susceptibility to asthma was associated with an increased risk of frailty in the discovery stage (odds ratio [OR] = 1.092, 95% confidence interval [CI] = 1.075–1.109, *P* = 5.00E-28), which was also validated in the replication stage (OR = 1.073, 95% CI = 1.052–1.096, *P* = 1.41E-11). Sensitivity analyses yielded consistent causal estimate, and no significant pleiotropy was found throughout the MR study.

**Conclusion:**

The present study demonstrated that asthma is causally associated with an elevated risk of frailty. Further studies are needed to elucidate the potential pathophysiological mechanisms between asthma and frailty.

**Supplementary Information:**

The online version contains supplementary material available at 10.1007/s40520-024-02906-4.

## Introduction

Frailty is a syndrome that leads to a decline in strength, endurance, and physiological functioning, resulting in increased vulnerability and a significant public health issue in the era of aging [1]. Its prevalence increases with age, and the incidence among females is higher than among males [2, 3]. Frailty is associated with adverse clinical outcomes such as falls, hospitalization, and mortality [4], severely affecting individuals’ quality of life and imposing a substantial global disease burden. Although various factors including genetic and environmental influences are believed to contribute to frailty, the underlying mechanisms remain undetermined [1, 5].

Asthma is the most common chronic respiratory disease in children and adults [6], characterized by various respiratory symptoms and airflow limitation [7]. Asthma has a significant incidence, mortality rate, and socioeconomic burden [8]. In 2016, the global number of asthma patients approached 339 million [9]. Observational studies have shown a higher risk of frailty in asthma patients, with a 2.19-fold increased risk of developing frailty among current asthma patients compared to non-asthma individuals in a 26 year follow-up study of adults residing in the community [10]. However, another study demonstrated no association between asthma and the elderly Fried frailty phenotype [11]. Therefore, the current evidence on the association between asthma and frailty remains inconclusive. Moreover, traditional observational studies cannot completely rule out reverse causality and residual confounding, making it challenging to infer causal relationships. Hence, further investigations are needed to determine whether asthma is causally related to the subsequent development of frailty.

Mendelian randomization (MR), utilizing genetic variant as an instrumental variable to assess causal relationships between the risk factor of interest and related diseases, can overcome inherent confounding biases in observational studies [12]. Here, we conducted a two-sample MR analysis, treating the genetic variant as an instrumental variable, to investigate the causal relationship between asthma and frailty. To validate the robustness of the MR results, we performed separate estimations using two independent asthma datasets.

## Methods

### Study design and data source

**Fig. 1:**
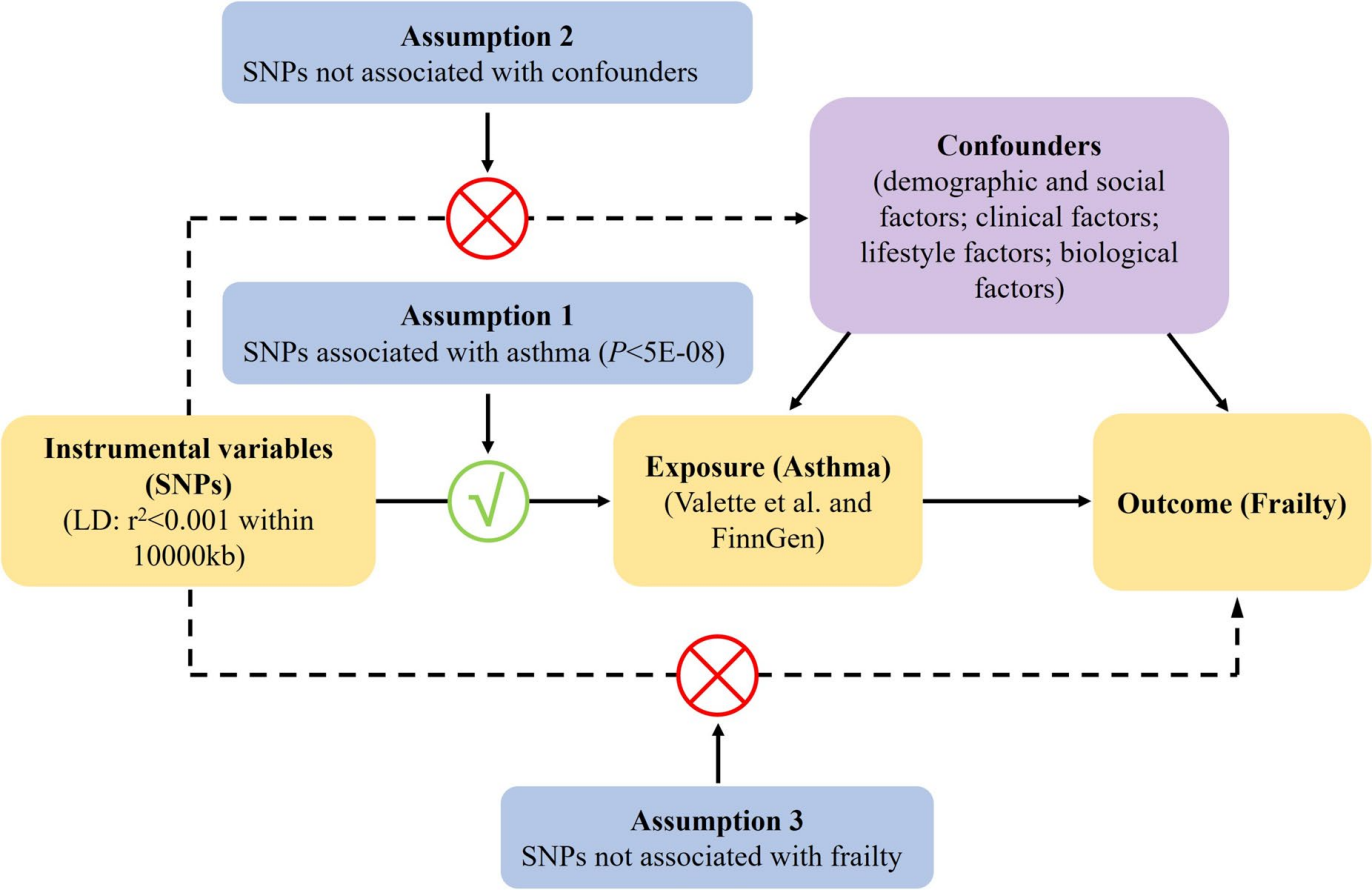
Design of the two-sample Mendelian randomization study. SNPs, Single nucleotide polymorphism; LD, linkage disequilibrium

We selected independent single nucleotide polymorphisms (SNPs) from genome-wide association studies as instrumental variables. The SNP used in this MR study need to satisfy the three key assumptions outlined in Fig. 1 [13]. The first assumption (relevance assumption) states that SNPs should be significantly associated with asthma. The second assumption (independence assumption) states that SNPs should be unrelated to confounding factors associated with both asthma and frailty, including demographic and social factors (advanced age, female gender, minority ethnicity, low education level, low socioeconomic status, living alone, loneliness), clinical factors (comorbidity and chronic diseases, obesity, malnutrition, cognitive impairment, depressive symptoms, polypharmacy), lifestyle factors (physical inactivity, low protein intake, smoking, increased alcohol consumption), and biological factors (elevated cytokines or CRP, deficiency in androgens or IGF-1, micronutrient deficiencies). The third assumption (exclusion restriction assumption) states that SNPs should affect frailty solely through their direct impact on asthma and should not be directly associated with frailty.

Two independent summaries of asthma data from the UK Biobank and the FinnGen Consortium were included in this MR study. The summary statistics from the UK Biobank comprised 56,167 asthma cases and 352,255 European ancestry controls [14], while the FinnGen Consortium contributed summary statistics with 30,309 asthma cases and 187,112 European ancestry controls (https://finngen.gitbook.io/documentation/data-download). We utilized the UK Biobank dataset for the discovery stage and the FinnGen Consortium dataset for the replication stage. The diagnosis of asthma was determined based on hospital records (ICD-9 or ICD-10 codes) or primary healthcare records, as well as self-reported asthma. Frailty, on the other hand, was often defined using frailty phenotype or the frailty index [15]. In our MR study, the assessment of frailty was based on the frailty index, which was calculated by aggregating 44–49 self-reported health deficits accumulated throughout the lifespan [15, 16]. The summary statistics for the frailty index were obtained from the most recent GWAS meta-analysis, which included 175,226 individuals of European ancestry. Detailed information regarding the study can be found in Table 1.

**Table 1:** Summary information of GWAS datasets in this Mendelian randomization study

Phenotype	Author	Year	Sample size (N)	SNP (N)	PMID	URL (Data Download)
Asthma (Discovery stage)	Valette et al	2021	408,442	34,551,291	34,103,634	https://doi.org/10.6084/m9.figshare.9204998
Asthma (Replication stage)	FinnGen	2021	217,421	16,380,048	NA	https://finngen.gitbook.io/documentation/data-download
Frailty	Atkins et al	2021	175,226	7,589,717	34,431,594	https://figshare.com/ndownloader/files/28842861

*SNP* Single nucleotide polymorphism, *N* Number

### Instruments selection

In the initial stage, the SNPs that surpassed the genome-wide significance threshold (*P* < 5E-08) were selected as instrumental variables (IVs). These IVs were then clumped based on linkage disequilibrium structure (European 1000 Genomes Project, r^2^ < 0.001 within 10,000 kb). Additionally, in preparation for the MR analysis, the SNPs associated with frailty with *P* values below 5E-05 were excluded from the IVs. We also employed the PhenoScanner v2 (http://www.phenoscanner.medschl.cam.ac.uk/) tool to check variants associated with the aforementioned confounding factors (*P* < 1E − 05). Variants correlated with these confounding factors were also excluded from the IVs [17]. (Supplementary Table S1). To ensure that all relevant exposure factors and outcome alleles were on the same causal pathway, efforts were made to harmonize the effects of these instrumental SNPs. All harmonized SNPs for each exposure-outcome pair were documented in the Supplementary information.

### Mendelian randomization analysis

The TwoSampleMR package (version 0.5.6) was employed for the current MR analysis. The inverse variance weighted (IVW) method was utilized as the default approach for estimating the causal relationship between asthma and frailty. IVW method is considered the most reliable when there is no evidence of directional pleiotropy in the selected IVs (MR-Egger intercept *P* > 0.05) [18]. A significance threshold of *P* < 0.05 was adopted to establish statistical significance. Additionally, MR-Egger, weighted median, and weighted mode methods were employed to validate the causal impact of the exposure on the outcome. Furthermore, we employed the Mendelian Randomization Pleiotropy RESidual Sum and Outlier (MR-PRESSO) analysis and Radial MR methods to detect SNPs with pleiotropic outliers (*P* < 0.05) [19, 20]. Upon identification of potential outliers, they were discarded, and we repeated the IVW estimation to assess the robustness of our results.

### Sensitivity, heterogeneity, and power analysis

We conducted sensitivity analyses to further ensure the robustness of our MR analysis results. Cochran’s Q statistic was employed to assess heterogeneity among SNPs. The MR-Egger intercept was used to test for the presence of directional horizontal pleiotropy in our MR analysis. Leave-one-out analysis was performed to examine whether the causal effect was driven by a single SNP. To assess the strength of each instrumental variable, we computed the *F*-statistic for each SNP, as previously described [21]. An *F*-statistic value greater than 10 indicates strong instrument strength and minimal bias due to sample overlap [22].

## Results

### Primary results

We successfully extracted 72 SNPs that were associated with asthma in the UK Biobank dataset. Among them, one SNP (rs13099273) was excluded from the analysis due to its palindromic nature, and four SNPs (rs10486391, rs12165508, rs1689510, rs4739738) were removed due to their association with confounding factors mentioned above. Therefore, the final MR study included 67 SNPs. In our analysis, the *F*-statistics values ranged from 29.8 to 251.5, surpassing the conventional threshold of 10, indicating the strong predictive potential of these instruments for asthma.

**Fig. 2:**
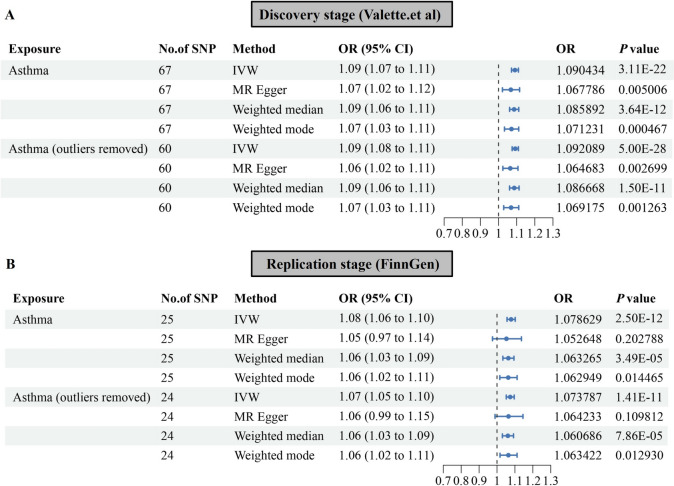
Forest plots of Mendelian randomization analyses show the causal effect of asthma on frailty. Use four different methods, including IVW, weighted mode, weighted median, and MR Egger regression, to evaluate the causal impact of asthma on frailty. (A, B) showed the causal effects of asthma on frailty during the discovery stage and replication stage, respectively. IVW, inverse variance weighed

**Fig. 3:**
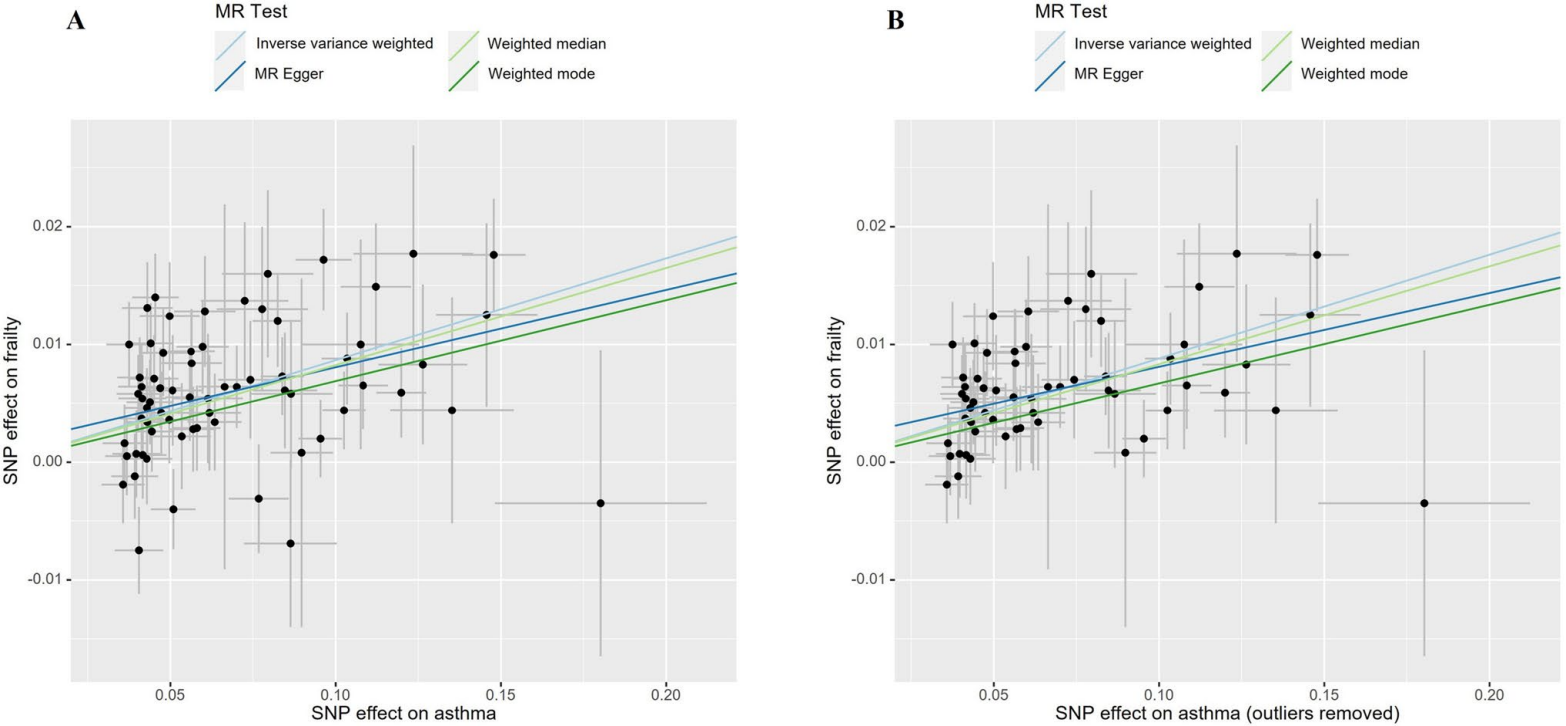
Scatter plot illustrating the potential impacts of SNPs on both asthma and frailty in the discovery stage. (A) showed scatter plots before removing outliers and (B) showed scatter plots after removing outliers

Using the IVW method, a significant positive correlation between genetically predicted asthma and frailty in the UK Biobank dataset (OR = 1.090, 95%CI = 1.072–1.110, *P* = 3.11E-22). Subsequent validation analyses using MR Egger, weighted median, and weighted mode methods yielded consistent results (Fig. 2A), with the scatter plot depicting the impact of each SNP on asthma and frailty (Fig. 3A). The consistency across the four MR models enhances the reliability of the causal relationship between asthma and frailty. Cochran’s Q test indicated heterogeneity in the assessment of the causal relationship between asthma and frailty during the discovery phase (Q value = 89.803, *P* = 0.027). However, there was no evidence of a significant intercept (intercept = 0.001495, *P* = 0.315), indicating the absence of observed pleiotropy. The summary of the results of the pleiotropy and heterogeneity tests is listed in Table 2. In addition, MR-PRESSO and Radial MR analyses were conducted. The MR-PRESSO test did not detect any outliers, while the Radial MR analysis identified seven outliers (rs13277355, rs174557, rs35570272, rs4099209, rs7423358, rs76493820, rs848) (Supplementary Fig. 1, Supplementary Table S2). We manually removed these outliers and repeated the IVW analysis, which still demonstrated the presence of a causal relationship (OR = 1.092, 95%CI = 1.075–1.109, *P* = 5.00E-28, Fig. 2A and 3B). Detailed information on the remaining SNPs is provided in Supplementary information. MR Egger, weighted median, and weighted mode methods showed consistent and significant estimates (Fig. 2A and 3B), and Cochran’s Q test did not indicate heterogeneity (*P* = 0.61). The results of the sensitivity analysis, omitting one instrument at a time, indicated that no single instrumental variable significantly influenced the overall causal effect (Supplementary Fig. 2 and 3). The funnel plot is depicted in Supplementary Fig. 4 and 5.

**Table 2:** Heterogeneity and sensitivity analysis of asthma on the risk of frailty

Exposure	Outcome (Frailty)				
	Method	MR-Egger intercept (*P*)	MR_PRESSO (*P*)	*F*-statistic	Cochran-Q (*P*)
Asthma (Discovery stage)	IVW	0.315	0.040	67.111	0.027
	MR-Egger				0.028
Asthma (Discovery stage with outliers removed)	IVW	0.171	0.859	68.266	0.826
	MR-Egger				0.852
Asthma (Replication stage)	IVW	0.523	0.388	54.981	0.344
	MR-Egger				0.315
Asthma (Replication stage with outliers removed)	IVW	0.805	0.618	55.361	0.580
	MR-Egger				0.523

*MR* Mendelian randomization, *IVW* Inverse-variance weighted, *MR_PRESSO* Mendelian randomization pleiotropy residual sum and outlier,* P* *P* value.

### Replication results

**Fig. 4:**
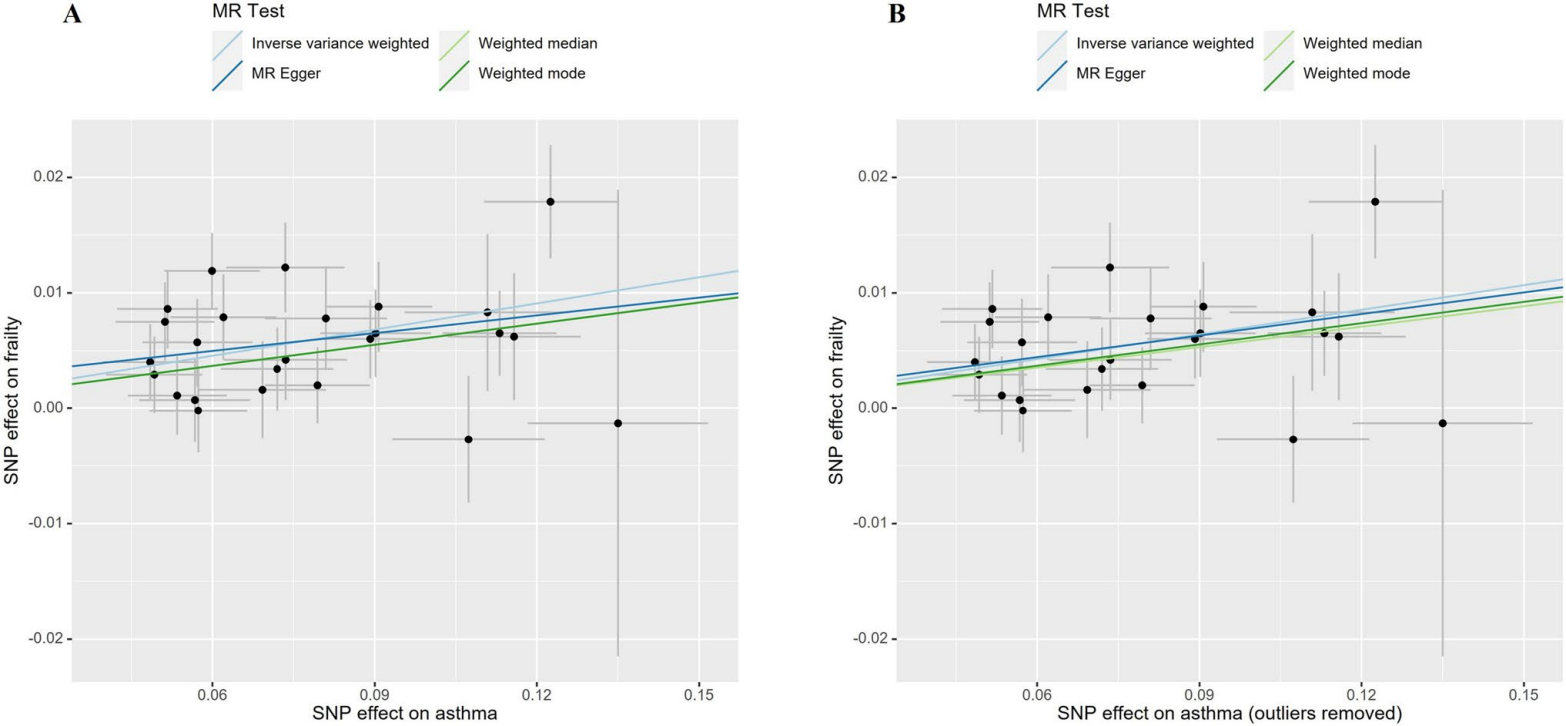
Scatter plot illustrating the potential impacts of SNPs on both asthma and frailty in the replication stage. (A) showed scatter plots before removing outliers and (B) showed scatter plots after removing outliers

In the FinnGen consortium dataset, 27 SNPs associated with asthma were extracted. Among them, one SNP (rs7936434) was excluded due to being palindromic in the harmonization process, and one SNP (rs8074437) was found to be related to confounding factors. Therefore, a total of 25 SNPs were included as instrumental variables for asthma in the MR analysis. In the replication phase, the estimates from the FinnGen dataset showed a directionally similar trend to the results from the UK Biobank (OR = 1.079, 95%CI = 1.056–1.101, *P* = 2.50E-12) (Fig. 2B), with the scatter plot once again illustrating the impact of each SNP on asthma and frailty (Fig. 4A). Cochran’s Q test and MR Egger intercept test did not detect heterogeneity and pleiotropy (Table 2). The MR-PRESSO test did not identify any outliers, while the Radial MR analysis detected one outlier (rs4277393) (Supplementary Fig. 6, Supplementary Table S2). After manually removing the outlier and repeating the IVW analysis, the causal relationship still remained (OR = 1.073, 95%CI = 1.052–1.096, *P* = 1.41E-11, Fig. 2B and 4B). Detailed information on the remaining SNPs is provided in Supplementary information. As shown in Supplementary Fig. 7 and 8, the Leave-one-out analysis revealed that when excluding each SNP one at a time, the residual effect of the remaining SNPs did not cross the zero line. This indicates that the results remained consistent and reliable. The funnel plot is depicted in Supplementary Fig. 9 and 10.

## Discussion

Frailty can be considered as an indicator of biological aging. With the intensification of global aging, preventing frailty remains a challenge. Understanding the causative factors of frailty contributes to a better comprehension of aging. Diseases such as cardiovascular and renal conditions, as well as depression, have been associated with frailty. However, the link between asthma, a component of chronic respiratory diseases, and frailty has not been widely explored. This study employed MR methods to investigate the causal relationship between asthma and frailty. Results indicate that genetically predicted asthma increases the risk of frailty, a conclusion consistently drawn from validation datasets related to asthma. Various MR analysis methods and sensitivity analyses further validate the reliability of this conclusion.

The positive correlation between asthma and frailty has been previously reported in traditional observational studies, supporting the current MR investigation. As mentioned above, a 26-year follow-up study involving community residents found that individuals with asthma had more than a twofold increased risk of frailty compared to non-asthmatic individuals [10]. However, most existing research on asthma and frailty has predominantly focused on epidemiological surveys of frailty in asthma patients, without assessing the causal relationship between the two. Earlier studies reported a median prevalence of frailty among asthma patients at 9.5%, ranging from 6% to 14.5% [23]. Another cross-sectional study covering 34,403 eligible participants from the National Health and Nutrition Examination Survey (NHANES) between 1999 and 2018 found a frailty incidence of 32.3% among asthma patients, with a 1.99-fold higher frailty incidence in asthma patients with concurrent chronic obstructive pulmonary disease (COPD) compared to those with asthma alone [24]. A cross-sectional survey of 224,142 individuals aged 60 and above across 31 provinces/autonomous regions/direct-controlled municipalities in mainland China revealed a high frailty prevalence of 35.8% among elderly asthma patients [25]. Clearly, the incidence of frailty in asthma patients and the causal relationship between the two warrant clinical attention. However, inherent limitations in observational studies, such as small sample sizes, unavoidable confounding factors, and the impact of reverse causation, hinder a definitive determination of the causal relationship between asthma and frailty. Our study employed genetic variants as instrumental variables to assess the causal effects between asthma and frailty, thereby overcoming the limitations inherent in observational studies. Ultimately, it concludes that genetically predicted asthma increases the risk of frailty. This further elucidates the causal correlation between asthma and frailty, emphasizing the routine assessment of frailty in the treatment of elderly asthma patients.

The current mechanisms underlying the association between asthma and frailty remain to be conclusively determined. Inflammation stands out as one of the reported biological mechanisms linking the two, with several common inflammatory markers identified in individuals with asthma and frailty [26–28]. Furthermore, asthma may interact with other chronic conditions associated with frailty to influence its manifestation. Existing research underscores the growing recognition of the impact of obesity on the diagnosis, management, and exacerbation severity of asthma, positioning it as a modifiable risk factor in asthma care [29]. The established relationship between obesity and frailty accentuates the potential increased risk of frailty in asthma patients due to suboptimal management of obesity. Additionally, the use of corticosteroids by asthma patients may contribute to the occurrence of frailty. A study investigating asthma patients aged 60 and above revealed a frailty prevalence of 37%, with a higher cumulative lifetime exposure to oral corticosteroids correlating with an elevated incidence of frailty [30]. Hence, emphasis is placed on the critical importance of minimizing oral corticosteroid exposure to prolong the healthy lifespan of individuals with asthma.

This study possesses several strengths, with the foremost being the utilization of a MR design. The MR design minimizes the impact of residual confounding factors and strengthens causal inferences regarding the association between asthma and frailty. The causal link between asthma and frailty was validated in two independent asthma cohorts, and the high consistency in results reduces the likelihood that the observed association is a chance finding. The large sample size of the MR study enhances the statistical power of the research. Additionally, the analysis in this study was restricted to individuals of European ancestry, reducing potential bias stemming from racial structural differences but limiting the generalizability of our findings to other populations.

We must acknowledge certain shortcomings in our study. Primarily, participants exclusively hail from European ancestry. While this choice serves to mitigate bias arising from racial disparities, it concurrently imposes constraints on the generalizability of our study findings to other populations. Secondly, there was heterogeneity observed in the primary results section during the initial MR analysis. However, this heterogeneity markedly diminished after the removal of outliers, and the secondary analysis of MR results closely approximated the initial findings. Furthermore, although *F*-statistics indicate the absence of weak instrumental variables, certain results might be influenced by the limitations of statistical power. Finally, the GWAS for the frailty index includes some participants from the UK Biobank, potentially causing overlap (not exceeding 34%) in the discovery phase and introducing possible bias. However, according to Burgess’s [21] estimation, the potential bias from a 30% sample overlap in this study is less than 0.1%. Therefore, it can be inferred that the bias introduced by sample overlap in the MR results is within an acceptable range. Additionally, the consistent conclusions in the validation phase further corroborate the stability of the MR results. Nevertheless, our study employed a series of rigorous statistical methods to minimize potential bias resulting from heterogeneity and pleiotropy.

## Conclusion

The present MR study suggests an association between genetically predicted asthma and the risk of frailty. Further investigation is warranted to elucidate the potential causal mechanisms underlying the impact of asthma on frailty.

## Supplementary Information

Below is the link to the electronic supplementary material.

Supplementary file1 (XLSX 28 KB)

Supplementary file2 (DOCX 1181 KB)

## Data Availability

No datasets were generated or analysed during the current study.
