# Supplementary material for: The causal association between asthma and the risk of frailty: A two-sample Mendelian randomization study
Source: Aging Clin Exp Res. 2024 Dec 27;37(1):10. doi: 10.1007/s40520-024-02906-4 (PMC11671423; doi:10.1007/s40520-024-02906-4)

**Title: The causal association between asthma and the risk of frailty: A two-sample Mendelian randomization study**

**Journal name: Aging Clinical and Experimental Research**

| **Supplementary Table S1.** SNP information associated with confounding factors identified by PhenoScanner | | | |
| --- | --- | --- | --- |
| SNP | Associated phenotype | *P* value | Pubmed ID |
| Discovery stage | | | |
| rs10486391 | Waist circumference adjusted for physical activity | 4.72E-06 | 28448500 |
| rs12165508 | Smoking | 7.47E-06 | UKBB |
| rs1689510 | Alcohol intake frequency | 6.94E-06 | UKBB |
|  | Years of educational attainment in females | 6.45E-07 | 27225129 |
|  | Years of educational attainment in males | 9.10E-07 | 27225129 |
|  | Years of educational attainment | 6.54E-12 | 27225129 |
| rs4739738 | Years of educational attainment | 4.21E-06 | 27225129 |
| Replication stage | | | |
| rs8074437 | Vitamin and mineral supplements: vitamin C | 7.22E-06 | UKBB |

| **Supplementary Table S2.** Detailed information on outliers detected by Radial MR analysis | | |
| --- | --- | --- |
| SNP | Q_statistic | p.value |
| Discovery stage |  |  |
| rs13277355 | 9.777065 | 0.001767022 |
| rs174557 | 6.601006 | 0.010192117 |
| rs35570272 | 6.332758 | 0.011852782 |
| rs4099209 | 4.47869 | 0.034320012 |
| rs7423358 | 4.925149 | 0.026468527 |
| rs76493820 | 4.617358 | 0.031649948 |
| rs848 | 4.810251 | 0.028290924 |
| Replication stage |  |  |
| rs4277393 | 4.780653 | 0.0287812 |

**Supplementary Fig. 1** Outlier map detected by Radial MR method in the discovery stage.


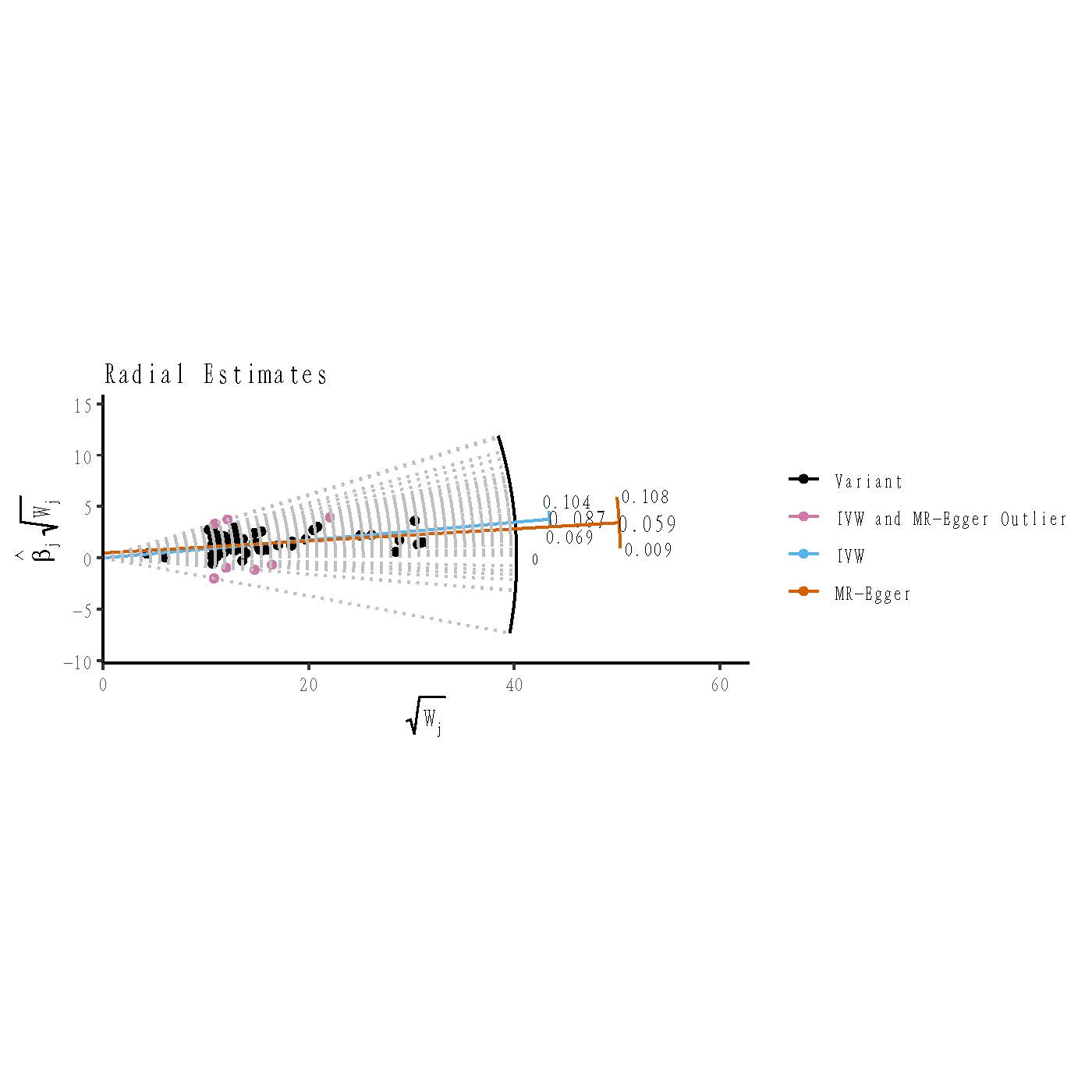


**Supplementary Fig. 2** Forest plots depicting the results of sensitivity analyses using a leave-one-out approach to assess the influence of asthma on frailty in the discovery stage without outliers removed.


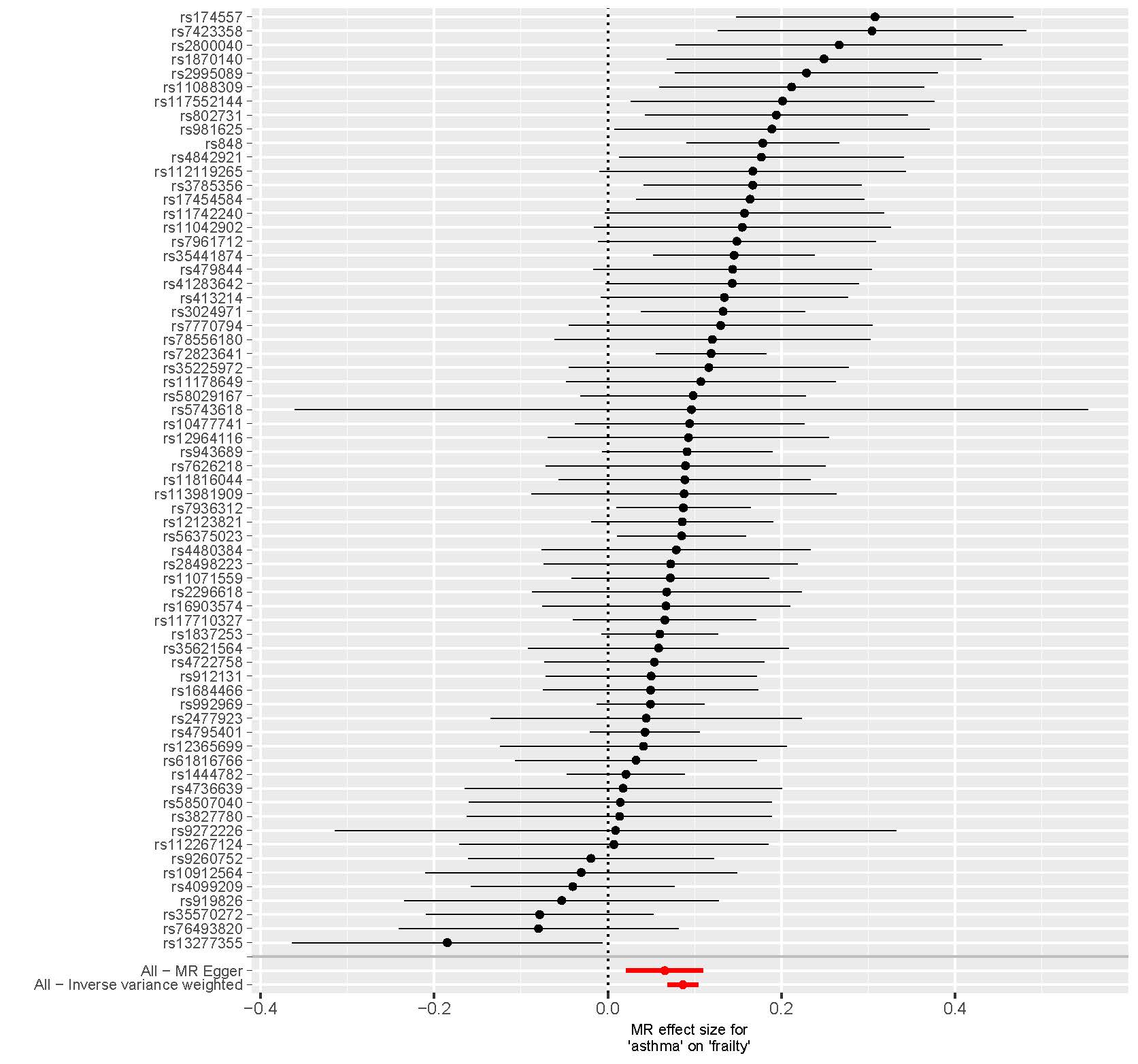


**Supplementary Fig. 3** Forest plots depicting the results of sensitivity analyses using a leave-one-out approach to assess the influence of asthma on frailty in the discovery stage with outliers removed.


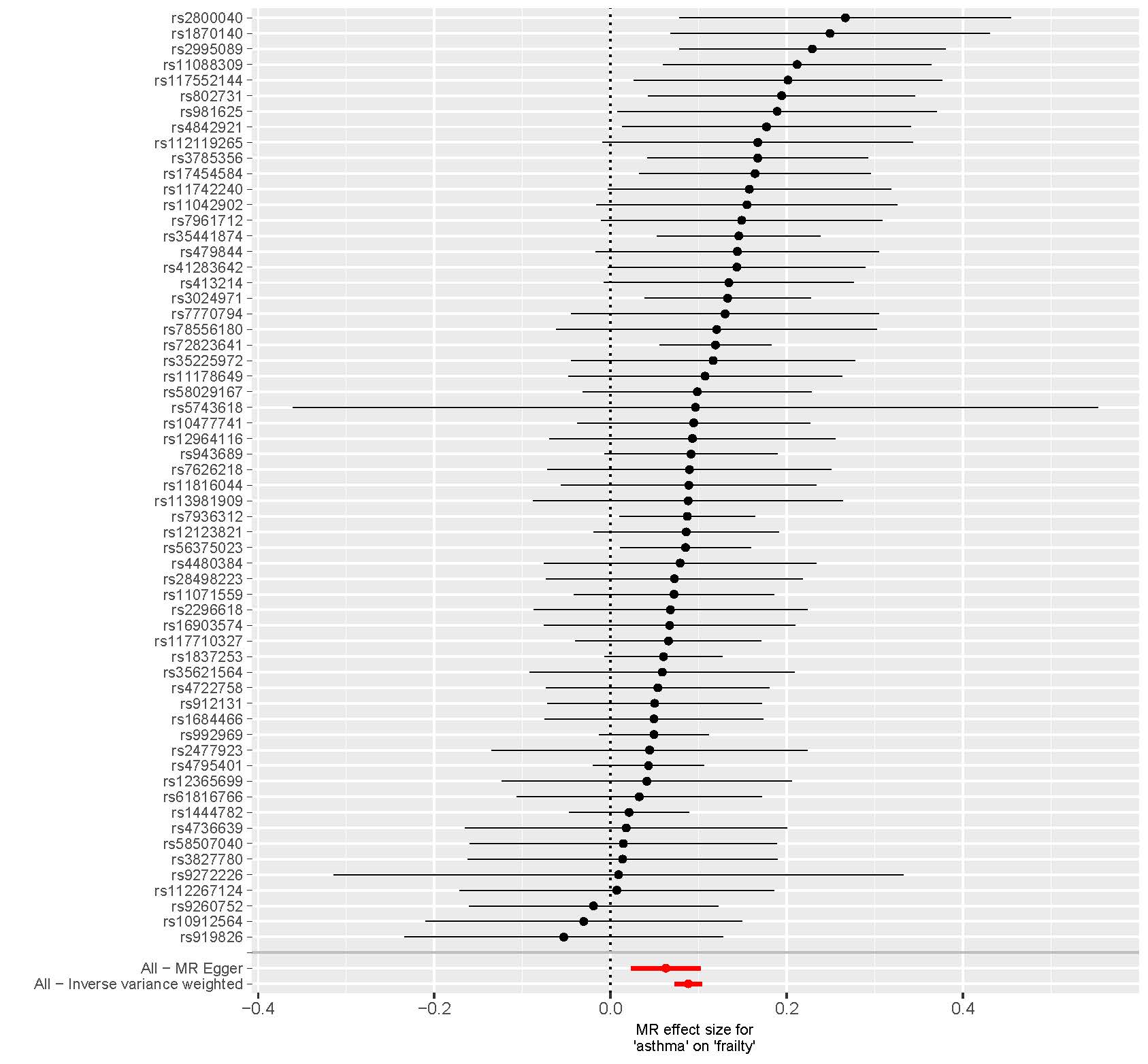


**Supplementary Fig. 4** Funnel plot for frailty regarding asthma in the discovery stage without outliers removed.


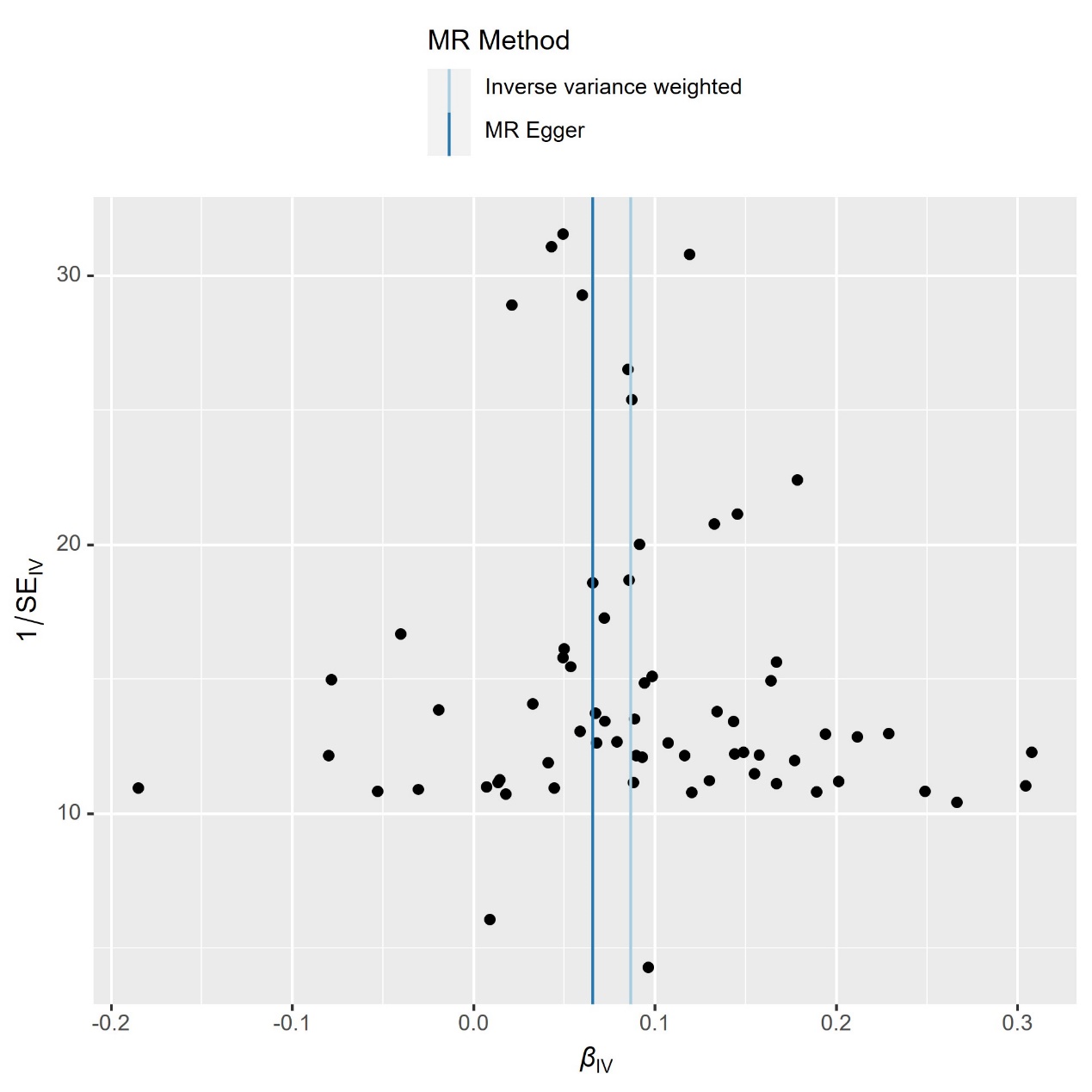


**Supplementary Fig. 5** Funnel plot for frailty regarding asthma in the discovery stage with outliers removed.


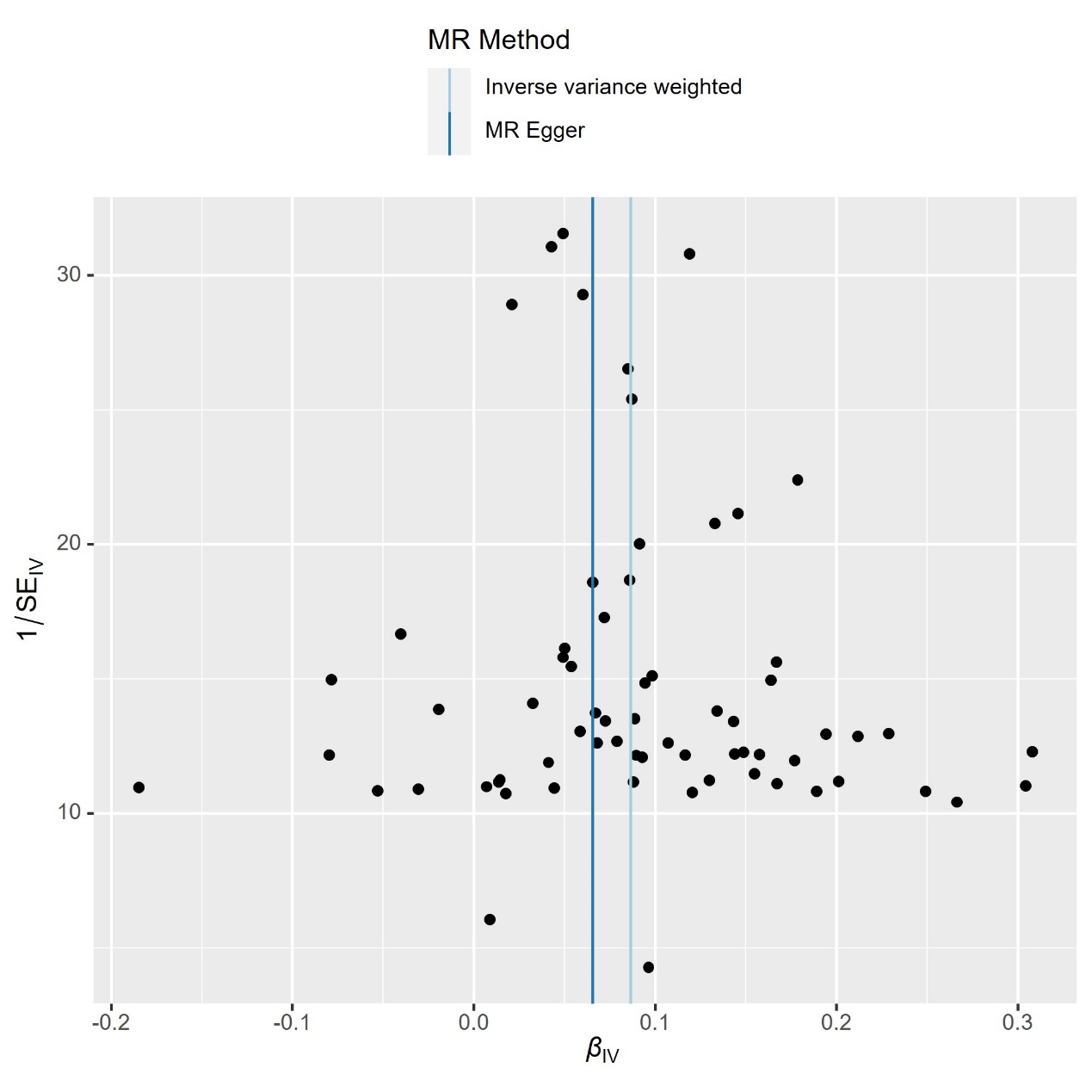


**Supplementary Fig. 6** Outlier map detected by Radial MR method in the replication stage.


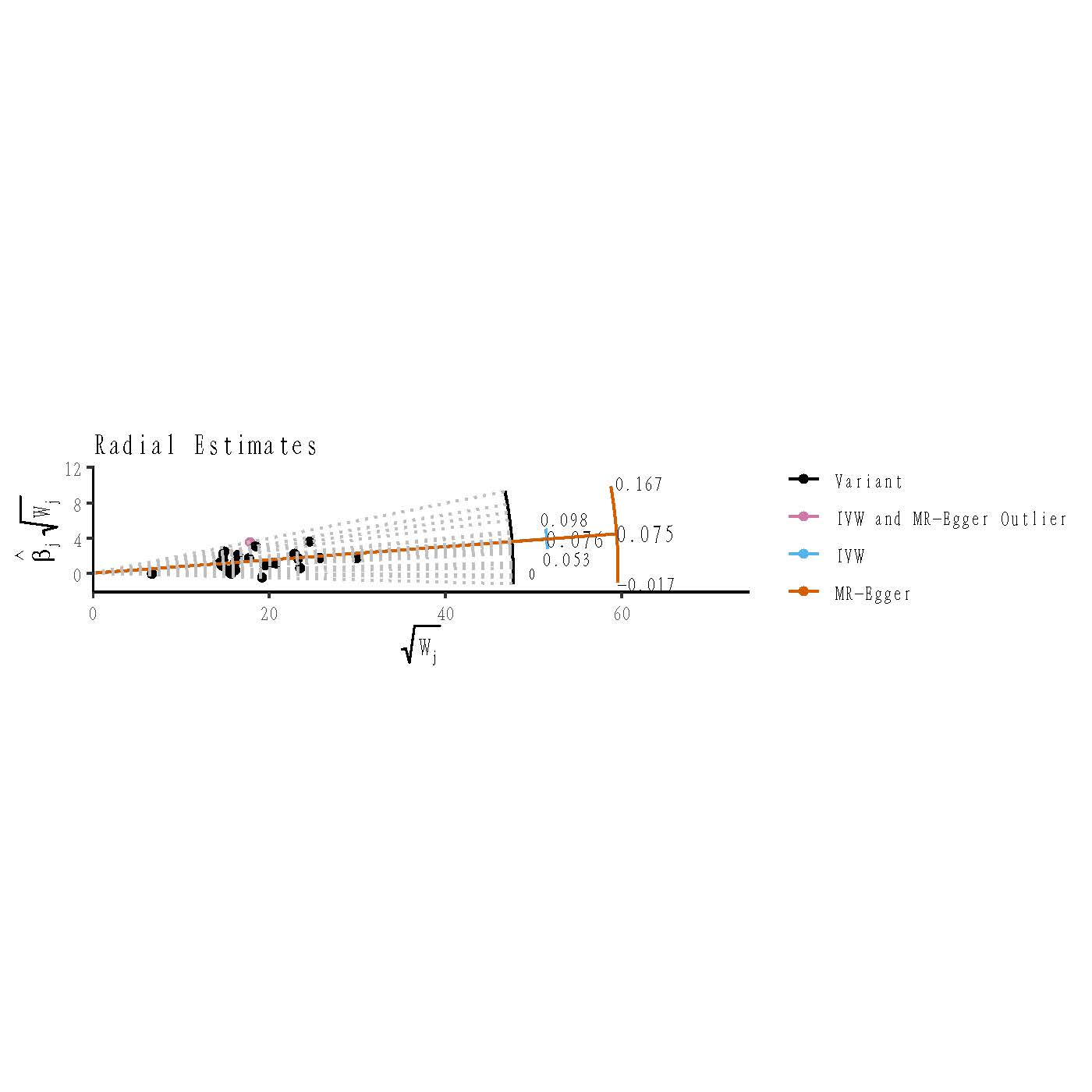


**Supplementary Fig. 7** Forest plots depicting the results of sensitivity analyses using a leave-one-out approach to assess the influence of asthma on frailty in the replication stage without outliers removed.


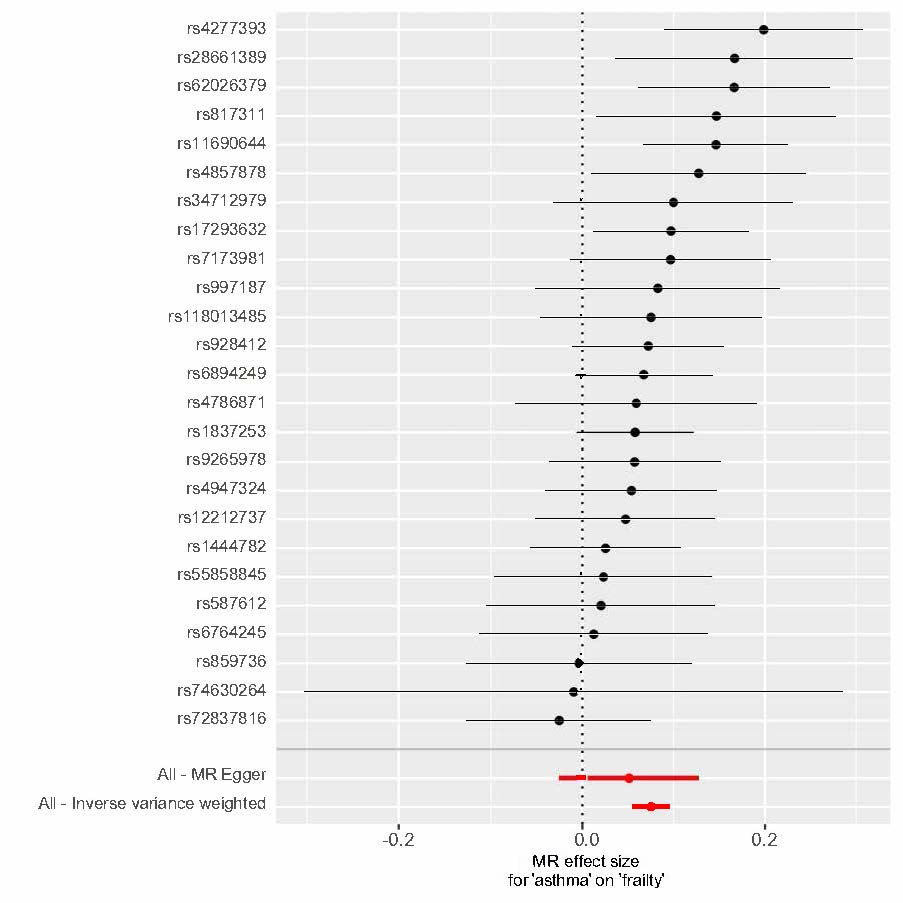


**Supplementary Fig. 8** Forest plots depicting the results of sensitivity analyses using a leave-one-out approach to assess the influence of asthma on frailty in teh replication stage with outliers removed.


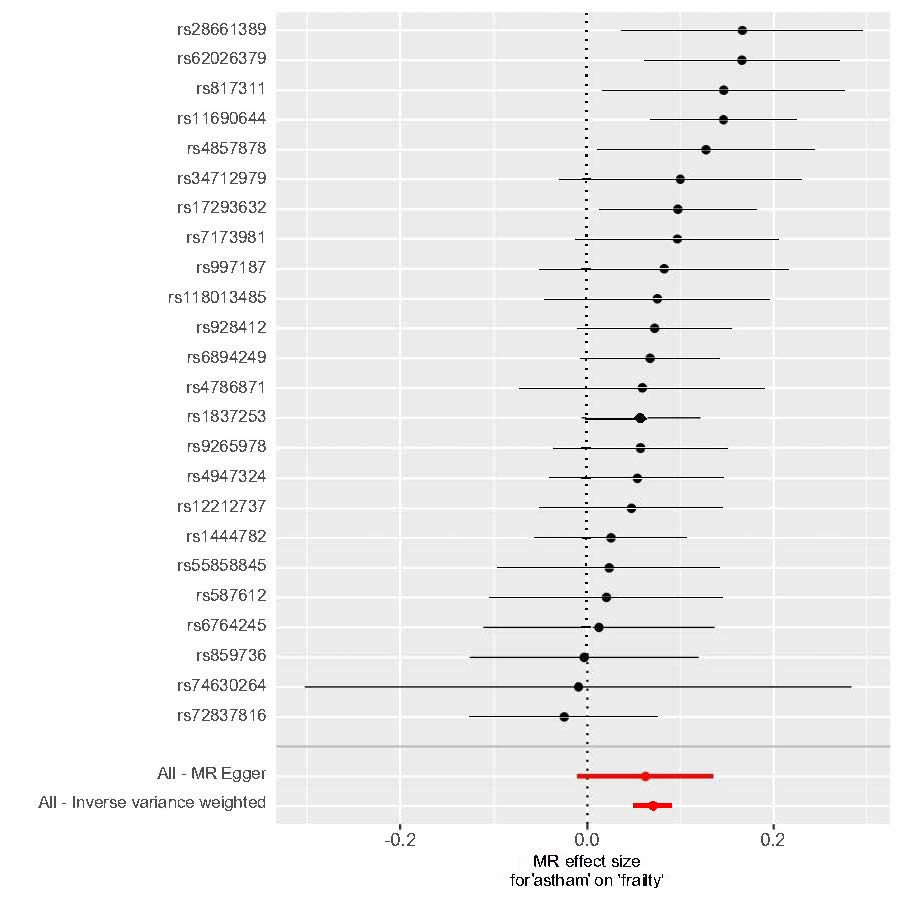


**Supplementary Fig. 9** Funnel plot for frailty regarding asthma in the replication stage without outliers removed.


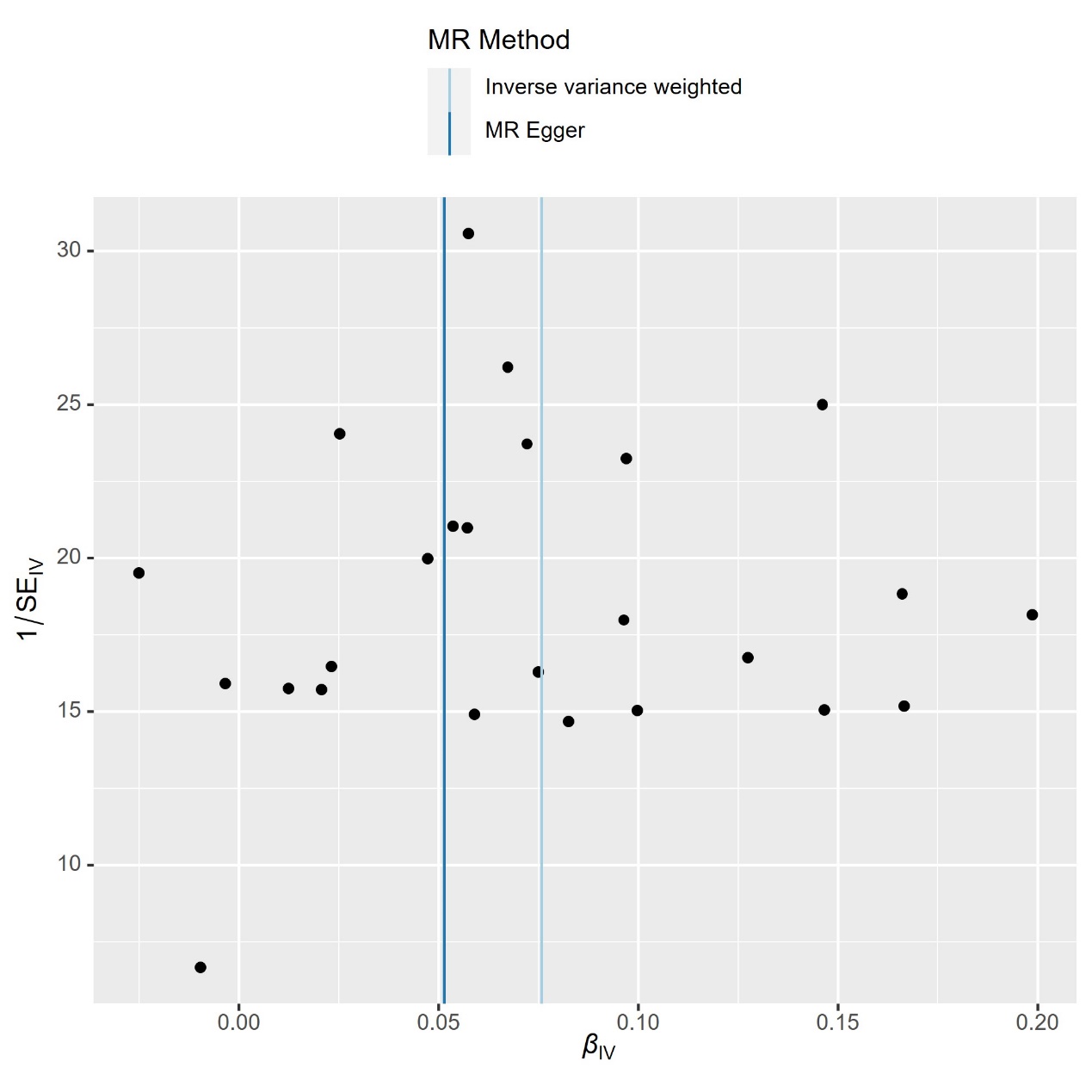


**Supplementary Fig. 10** Funnel plot for frailty regarding asthma in the replication stage with outliers removed.


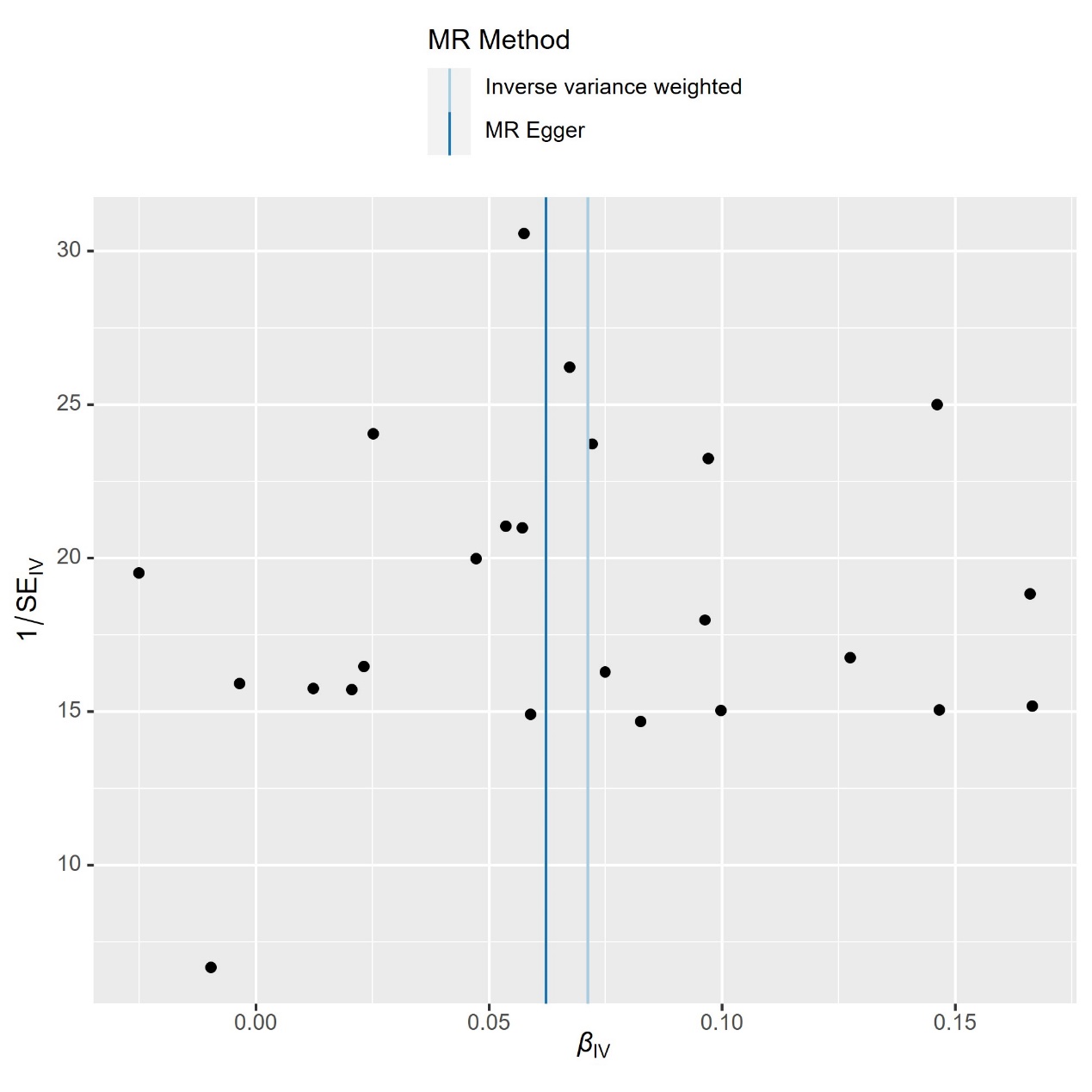

Supplement: Supplementary file 2 — Supplementary file2 (DOCX 1181 KB) [file 40520_2024_2906_MOESM2_ESM.docx]
